# Nurses’ perspectives on pain management in pediatric care: systematic review and meta-synthesis

**DOI:** 10.3389/fped.2026.1814470

**Published:** 2026-05-08

**Authors:** Majd Elmobasher, Hui Tao, Jing Guo, Issam AbuQeis, Abeer Teeti, Yu Wang, Ma Fang

**Affiliations:** 1 School of Nursing, Kunming Medical University, Kunming, China; 2 Nursing Department, The First Affiliated Hospital of Kunming Medical University, Kunming, China; 3 School of Basic Medicine, Kunming Medical University, Kunming, China; 4 Department of Epidemiology, School of Public Health, Kunming Medical University, Kunming, China

**Keywords:** barriers, meta-synthesis, nurses' perspectives, nursing practice, pain assessment, pediatric pain management, qualitative research

## Abstract

**Background:**

Pain remains one of the most frequently misinterpreted, underdiagnosed, and inadequately managed clinical conditions in pediatric populations. An estimated 50%–70% of pediatric patients experience undertreated pain, which can have serious negative effects on their overall health and well-being. Our study aims to identify, appraise, and synthesize qualitative studies exploring nurses’ perspectives on pain management for pediatric patients to develop a conceptual understanding of facilitators and barriers from the nurses’ perspective.

**Methods:**

This review was registered with PROSPERO (CRD420251034205). A comprehensive literature search was conducted across CINAHL, PubMed, and Nursing journals for English-language studies. The SPIDER framework was used to guide the selection of qualitative studies. Inclusion and exclusion criteria were applied following the PRISMA 2020 guidelines, resulting in the inclusion of nine studies in the final synthesis. Confidence in the synthesized findings was assessed using the GRADE-CERQual (Confidence in the Evidence from Reviews of Qualitative Research) approach.

**Results:**

Analysis of nine qualitative studies identified five interconnected themes shaping nurses’ perceptions of pediatric pain management. *Being Defeated by Pain* reflected nurses’ emotional and professional struggles with persistent or poorly managed pain. *Family Participation is a Necessity* emphasized the vital role of caregivers in assessment and decision-making. *Evidence-Based vs. Experience-Based Pain Assessment* revealed tension between adherence to clinical guidelines and reliance on personal judgment. *Pharmacological vs. Non-Pharmacological Interventions* highlighted challenges in selecting appropriate treatment strategies within practical constraints. *Culture-Related Pain Management* illustrated how cultural beliefs influence both pain assessment and intervention. Collectively, these themes underscore the complex, multifaceted nature of pediatric pain care from the nursing perspective. The GRADE-CERQual assessment indicated high confidence in the findings for “Being Defeated by Pain” and “Pharmacological vs. Non-Pharmacological Interventions,” and moderate confidence for the remaining three themes.

**Conclusions:**

Nurses face multifaceted challenges in pediatric pain management. Targeted, culturally sensitive strategies are needed to enhance nursing competence and quality of care.

## Background

Pain remains one of the most frequently misinterpreted, underdiagnosed, and inadequately managed clinical conditions, particularly among pediatric populations (1). A synthesis of inpatient studies indicates a 95% prevalence of iatrogenic and disease-related pain in hospitalized children, stemming from procedural, diagnostic, and pathological factors (2). Furthermore, qualitative analyses consistently identify pain and procedural discomfort as the most distressing aspects of pediatric hospitalization across multiple studies (3). Untreated or undertreated pain can negatively impact various aspects of a child's well-being, including psychological state (mood disturbances), physiological functioning (disrupted sleep and appetite), and social participation (reduced school attendance and academic engagement). Evidence confirms that untreated childhood pain contributes to long-term morbidity and familial economic strain (1). Moreover, chronic pain may limit participation in physical activities and peer interactions, potentially leading to long-term functional disability and a decreased quality of life (4).

Although there are some evidence-based guidelines for pediatric pain management, insufficient pain management still occurs. Unsatisfactory pain management in children remains a widespread clinical concern, particularly in postoperative settings. Evidence indicates that 50%–70% of pediatric patients experience undertreated pain after surgery, with significant variability in analgesic administration (4). Assessment challenges further complicate care, as 20%–40% of young children cannot self-report pain reliably, necessitating observational tools like the FLACC scale, which remains inconsistently adopted (5). The consequences are clinically significant: poorly managed pain correlates with long-term hyperalgesia, delayed recovery, and heightened parental distress (6). As frontline providers, nurses play a pivotal role in pain management in childcare. Nurses’ perceptions of pain management encompass their clinical judgments, attitudes, and decision-making processes regarding pain assessment and intervention, which can influence the implementation of pain management.

Nurses’ perspectives on children's pain management revealed complex cognitive frameworks shaping assessment and intervention approaches. A pivotal study examining pediatric nurses’ cognitive representations (CRs) of children's pain revealed that while 65% of nurses incorporated children's self-reports in their assessment approach, 80% relied on behavioral manifestations, with only 50% integrating both methods, suggesting a preference for observable indicators over subjective reports. For pain management strategies, 75% of nurses identified pharmacological approaches as central to their practice, while 60% recognized non-pharmacological interventions, and only 35% acknowledged family involvement as a component of comprehensive pain management (7).

Additional research has explored the beliefs and perceptions of healthcare professionals regarding pediatric pain, revealing that nursing perspectives on pediatric pain are influenced by a combination of clinical experience, formal education, and individual belief systems, which collectively shape assessment priorities and intervention decisions. The interrelationship between how nurses conceptualize pain and their subsequent medication administration practices emphasizes the fundamental importance of comprehending these perspectives to enhance pain management outcomes in pediatric settings (8).

Therefore, this meta-synthesis aimed to identify, appraise, and synthesize qualitative studies exploring nurses' perspectives on pain management for pediatric patients to develop a conceptual understanding of facilitators and barriers from the nurses' perspective.

## Methods

### Design

We used meta-aggregation methodology to conduct a systematic review and meta-synthesis (integrating qualitative findings). The study followed five key stages: (1) Define the research question, objectives & scope. (2) Conduct a systematic literature search of relevant studies. (3) Perform a comprehensive quality appraisal of the selected studies. (4) Extract data. (5) Synthesize Findings (Meta-aggregation). The study protocol was registered in the PROSPERO (Preferred for Health-Related Reviews) database (DOI: CRD420251034205) on April 16, 2025, to enhance transparency and minimize bias.

### Data sources and searches

Three authors independently searched the following databases: CINAHL (nursing and allied health database), PubMed, and Nursing Journal to ensure comprehensive coverage of nursing literature. All identified search terms were systematically categorized according to the predetermined eligibility criteria, which were conceptualized using the SPIDER framework (Sample, Phenomenon of Interest, Design, Evaluation, and Research type (9), for qualitative research.

1. S (sample)**:** Registered nurses and nurse practitioners, providing direct patient care.
2. PI (phenomenon of interest)**:** Subjective experiences, clinical decision-making processes, and perceived barriers/facilitators in assessing and managing pain in children.
3. D (design): Primary qualitative studies including phenomenology, grounded theory, or ethnography designs.
4. E (evaluation)**:** Thematic outcomes related to practice challenges, institutional influences, or interdisciplinary dynamics (10).
5. Research type: Qualitative study.

**Figure 1:**
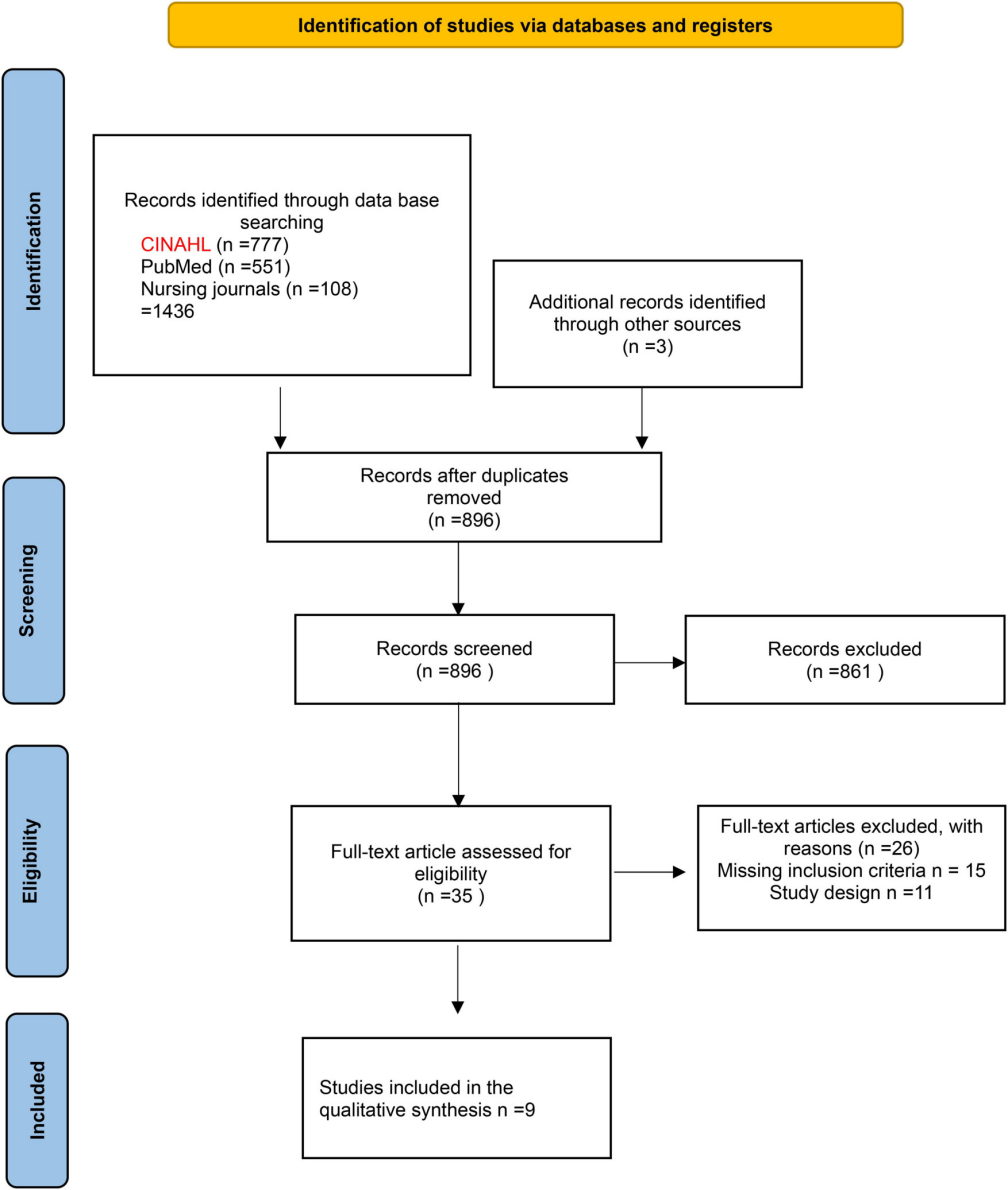
Study selection flow diagram. (Supplemetary Table S2 for details and rationale for the excluded studies).

In addition, we used footnote chasing, citation searching, hand searching, the journal runs, and author searching to ensure a comprehensive search of studies that met our inclusion criteria; the detailed search was reported in Figure 1.

### Study selection

The inclusion criteria adopted were qualitative primary studies published in English (2010–2024), primary qualitative data on nurses' perspectives, studies focusing on pediatric populations, and mixed-methods studies with clear qualitative data. The exclusion criteria were mixed-methods studies without separable qualitative data and Conference abstracts/theses without full text. Two authors, Hui Tao, and Ma Fang, independently reviewed the studies. After identifying the records in the databases, we removed all duplicates.

### Data extraction and quality assessment

Data from included studies were independently extracted by two authors, Hui Tao, and Ma Fang, and organized in an ad-hoc synoptic table, including a year of publication, country (setting), aim, participants, data collection, data analysis, and the qualitative findings summarizing the participants' experience (Table 1). Our analysis utilized the Critical Appraisal Skills Program (CASP) tool (11), a standard assessment framework in qualitative health studies, to critically appraise the methodological rigor (risk of bias), credibility (confidence in findings), and relevance (applicability) of included studies, with explicit documentation of methodological limitations during evidence synthesis.

**Table 1:** Characteristics of included studies.

Study author(S), Year	Study design	Study purpose	Sample size & characteristics	Methodology	Key findings/themes	Study context/setting	Key quotes (if applicable)	Data analysis
Mediani et al. (2017) (19) (Indonesia)	Descriptive qualitative	To explore Indonesian nurses’ perceptions of and experiences with pain management focussed on perceptions of barriers to pain management	*N* = 37 M/F = 7/30. Age = 25–60 years Characteristics: more than three years of experience, full time work	Semi-structured, in-depth interviews	(a) imbalance in nurse–patient ratios, (b) lack of sufficient education/training, (c) lack of organizational support, (d) lack of professional autonomy and (e) feeling powerless	Two states in West Java Province, Indonesia. Data collection occurred between October 2010 and December 2011	I can not focus on caring for paediatric patients … I have many tasks to complete on this shift … I am not able to deliver effective pain care Our academic education at diploma and degree level does not prepare students to conduct pain assessment and management adequately because we have only received the knowledge in brief	Grounded theory
Aziznejadroshan et al. (2018) (20) (Iran)	Qualitative study	To explore the experience of nurses’ barriers to pain management in pediatric units	*N* = 19 Age = 29–49 years Characteristics: Work experience:5-24 years Educational attainment: bachelor (15) Master (4)	Interview	The five elicited themes of pain management are: (a)Inappropriate organizational structure. (b)inadequate nursing competency (c)interruption in pain relief activities. (d)individual characteristics of child and parents. (e) inefficacy of companions	Two educational pediatric hospitals namely amirkola pediatric hospital in babol and children’s medical center in tehran	“We have not enough time to use pain-assessment tools for large number of child.” “As pethidine is kept in the nursing office, access to it involves undergoing some procedures” “Like nurses, I have not seen any doctor who use pain assessment tool and issue orders based on it”	Conventional content analysis 1) Transcribing the whole interview immediately after it is done. (2) Reading the whole transcribed text to gain a general knowledge of its content. (3) Determining the meaning units and initial codes. (4) Categorizing similar initial codes in more comprehensive groups. (5) Determining the main theme of per category
Berihun et al. (2024) (21) (Ethiopia)	Mixed method study	To assess practice and factors associated with pediatrics pain management among nurses working in bahir dar city public hospitals	*N* = 8 M/F = 6/2. Characteristics: Age above 30 years Experiences in nursing: more than five year	Semi structured in-depth interview	Four main themes and eight sub-themes (a) Inadequate knowledge of pain assessment and pain management practice. (b) Inadequate professional commitment and satisfaction. (c) Organization-related factors. (d) Knowledge, culture, and economic status of the family-affected pediatrics pain management practice for hospitalized children	Public hospitals, amhara region, north west ethiopia	“If they have a fever, we will do cold compress. If elevation is done on trauma, we will do it. We do not do anything other than these. The rooms are not comfortable, they are overcrowded and this makes it difficult for us to provide the services we need.” “… Absence of tools, there is no updated protocol; science is new every time…	Qualitative data was analyzed through the thematic analysis using atlas ti version 7.0 software, major and subthemes were created from the text itself through repeated reading
Susanto et al. (2022) (22) (Indonesia)	Qualitative descriptive approach	Explored nurses’ experiences providing pain management in the pediatric unit	*N* = 15 Characteristics: Aged 26–47 years Participant: 4 from the pediatric general ward, 4 from the maternal-child ward, 3 from the vip ward and 4 from the picu all participants were female, muslim and had been working between 3 and 16 years, 10 of the nurses had nursing diplomas and 5 had bachelor’s in nursing. 6 of them were team leaders on the wards	One-to-one telephone interviews	Five themes emerged from the data: (a) Ways to assess pain vary (b) Working with colleagues. (c) Attitudes toward pain-relief strategies. (d) Enhancing parental understanding and involvement. (e) Desire to have age-specific pain management trainings for pediatric patients	Islamic teaching hospital in central java province, indonesia (Pediatric general, maternal-child, and vip wards, pediatric intensive unit (picu)	Assessment is number one.., we must do it for every patient here. whether there is pain or anxiety. it may be visible in the patient’s hemodynamics on the monitor. also nurses keep asking the patients whether they have pain or not, because that is the basis for taking further steps. during emergency conditions, we automatically give analgesics to reduce pain When i give pain medication (paracetamol) while reciting bismillahirrahmanirrahim (meaning, “in the name of allah, the most gracious and the most merciful”).. afterwards, the pain will definitely disappear and one can sleep. after 30 min, i come back to the patient to see their condition. usually, they go to sleep because they feel better	Content analysis
Parra-Giordan et al. (2020) (23) (Chile)	Descriptive qualitative design	To know the perception of nursing professionals regarding pain management in pediatric oncology people hospitalized during the second half of 2017	*N* = 6 Characteristics: female, age range (27 and 49)	A semi-structured interview	Category (a) Definition of pain (b) Assessment of pain (c) Pain care planning (d) Pain treatment	Hemato-oncology unit of the hospital de niños roberto del río	"It is like a “total pain” (..) it has many areas that affect “the whole life of the child,” that is why i consider it as “total pain” “They are the ones who know the child. there is no lack of the mother who says”, aunt, it’s strange, something is bothering you, do you think it's a pain? “,” let's try it” “When one manages to have an effective therapeutic relationship, and that is only by doing humanized care, they will have full confidence in you and also that you will be able to help them, and that reduces the burden of anxiety on the child”	Content analysis
Kusi Amponsah et al. (2020) (24) (Ghana)	A descriptive qualitative study	Identify and understand the nursing-related barriers to childrens pain management in the ghanaian context	*N* = 28 Characteristics: 24 female, 4 male Ages ranged from 24 to 38 years Work experiences an average of five years (range of six months to 14 years) obtained certificate (*n* = 5), diploma (*n* = 16), bachelors degree (*n* = 5), and masters degree (*n* = 2)	Individual And Group Interviews	Visual representation of identified pain assessment and management barriers. (a) Pain assessment barriers: Misconceptions on childrens pain. Lack of pain assessment tools (b) Pain assessment and management barriers Communication challenges in preverbal and nonverbal children. Insufficient education (c) Pain management barriers Insufficient number of nurses. Lack of prescriptive authority on analgesics	Five hospitals in ghana (pediatric care settings)	For the assessment, its kind of difficult… especially for those who can not verbalize that they are in pain because we have not received any training on how to assess pain, its kind of difficult but once you do not know that the child is in pain, how do you manage? hmmmm (participant 3, hospital a) The patient ratio to nurse ratio is not all that good… you would not have time you have a lot of work to do and here we are dealing with kids, we work with time so by the time a child is complaining of something, maybe you are doing something, you attend to another child so you can not get enough time to do the diversional therapy for the child	Each recorded interview was transcribed verbatim and checked for correctness by the participants and at least two members of the research team The codes were then combined to form larger meaningful units using deductive analytic techniques until the agreed themes were actively generated nvivo 12 plus software guided the management and analyses of the qualitative data
Silva et al. (2011) (25) (Brazil).	A descriptive and qualitative study	To describe the experiences of pain management from healthcare professionals working in intensive care units	*N* = 11 Inclusion criteria were: directly assist children, be part of the shift scale of the sector during the research and agree to participate	Semistructured interview	The following categories have emerged from interviews analysis: The meaning of hospitalized childrens crying. Pain evaluation by the nursing team Perceiving pain situation in hospitalized children. Minimizing hospitalized childrens pain	Pediatric sector of the teaching hospital clemente de faria (hucf)	“When children cry, I think they are feeling pain, or fear, or are their mothers, or because they are in an unknown place and, most of the times, they feel pain (E2)” “Pain due to the disease, in general with venous punc- tures, drug administration, dressings, procedures per- formed according to the disease (E3)” “In general we medicate and, depending on the type of pain, if it is local one may apply cold or warm packs depending on the situation and on the indication (E3)”	Content analysis
Bhattacharyya et al. (2024) (26) (UK)	A qualitative study	TO describe the experiences of pain management from healthcare professionals working in intensive care units	*N* = 30 Nurses and physicians. Inclusion criteria: worked as an icu nurse or physician (defined as medically qualified clinicians from any speciality background)	Face-to-face interview with audio recording	Five Main Themes Were Constructed: (a) Pain assessment in the icu (b) Resource availability (c) Importance of pain in icu population (d) The emotive experience of critical care staff when acting as pain practitioners (e) Complexity Of The Icu Population	8 Hospitals in Uk.	“I wasn't convinced his pain was real…he'd had a liver transplant with a history of drug addiction prior…I thought maybe he wasn't in as much pain as he was demonstrating outwardly that he was” – Senior Nurse 1 Interviewer: “Do you think your physician colleagues are aware of the CPOT?”. Interviewee: “Well, honestly speaking, not too much…CPOT ends up being only for nurses” – Junior Nurse 3	An inductive thematic analysis technique
Skog et al. (2020) (27) (Sweden)	A qualitative interview study	To increase the understanding of Swedish nurses’ views on the assessment of chil- dren’s pain, with a focus on pain scales	*N* = 12 Nurses working in children hospital, with varying professional experience	Stimulated recall interview(SRI)	One theme: Need for higher competencies and evidence Three categories: routines can enable pain assessment, trusting one’s own assessment of the whole picture, and pain assessment scales as an extra workload.	Children's wards in a hospital in southern Sweden	“It hurts, yes, it does. But if you want to get a completely clinical view and the clinical state of the patient, it’s not just the pain that you look at, but the whole picture, together with the background, the reason for the hospitalization, that is.” (Informant 2) It may be that we have too many tools, it's easily deprioritized, unfortunately.(Informant 12)	Qualitative content analysis A descriptive theme was used to describe the major thread of the data and answer the question

The CASP checklist (11) systematically evaluates ten methodological components: study rationale, qualitative design appropriateness, sampling framework, data collection rigor, reflexivity documentation, ethical adherence, analytical transparency, results validity, and knowledge advancement. A dichotomous scale (Y: yes/N: no/CT: cannot tell) appraises reporting quality for items 1–9, with item 10 requiring narrative synthesis of the study's impact (valuable or not valuable). Study quality was independently appraised by two reviewers, Hui Tao, and Ma Fang, with any discrepancies adjudicated through team discussion to reach a consensus.

### Data synthesis and analysis

The data synthesis and analysis process was conducted concurrently rather than sequentially (12). Following the PRISMA framework and JBI standardized methodology, two independent researchers (TH and MF) performed analytical steps simultaneously to ensure methodological rigor (13). Initial familiarization was achieved through repeated readings and line-by-line analysis of the included studies to identify key concepts. Findings were extracted and classified as “Unequivocal” (U), “Credible” (C), or “Not Supported” (NS), based on the strength of their evidentiary support (14). These findings were subsequently arranged into initial thematic groupings by aggregating those that exhibited comparable meanings or conceptual emphases. To ensure that the synthesis remained firmly grounded in participants' original accounts, a comprehensive audit trail was maintained, systematically connecting the primary data to the findings, the findings to their respective categories, and the categories to the synthesized themes (Table 2) (15). Through a process of abstraction and constant comparison, redundancies were reduced, and five higher-order themes were developed to provide a comprehensive interpretation of the phenomenon (16). Conflicting findings were explored through iterative analysis and preserved as tensions within the themes to maintain analytical depth. Finally, the confidence in each synthesized theme was appraised using the GRADE-CERQual profile (Table 3), evaluating four key domains: methodological limitations, coherence, adequacy of data, and relevance (17, 18). To ensure trustworthiness, we implemented five key strategies: (1) assembling a multidisciplinary research team, (2) conducting a systematic literature search, (3) employing multiple coders at all stages, (4) refining themes iteratively through regular team discussions, and (5) maintaining comprehensive audit trails. Analytical decisions emerged from collaborative “think-aloud” discussions, with any discrepancies resolved by consensus-building.(12)

**Table 2:** Example of coding, category development, and final themes.

Initial Codes	Categories	Theme
Workload (49) Imbalance in nurse–patient ratios (20) Lack of professional autonomy (20)	Systemic and organizational barriers	. Being Defeated by Pain
Lack of training and unprepared nurses (19) Lack of resources and protocols (21)	Educational and competency deficits	

**Table 3:** CERQual evidence profile.

Review finding	Studies contributing to the review finding	Methodological limitations	Coherence	Adequacy	Relevance	CERQual assessment of confidence in the evidence	Explanation of CERQual assessment
1. Being Defeated by Pain	(20),(19),(21),(22)	Low methodological limitations. The studies contributing to this finding (primarily 19, 20, 24) were assessed as having high methodological quality using the CASP tool. The study notes some minor inconsistencies in data collection across the entire body of evidence, but the core studies for this theme are sound.	No or very minor concerns about coherence. The finding is highly coherent, with clear alignment between the data from the primary studies and the synthesized theme. The concept of being “defeated” is well-supported by participant quotes reflecting struggles with systemic constraints, professional limitations, and psychological distress.	Minor concerns about adequacy While the data are rich and provide a good understanding of the phenomenon, the theme is primarily supported by a subset of the included studies. Broadening the evidence base with more diverse settings could further strengthen this finding.	Minor concerns about relevance (6 studies with direct relevance, 2 studies with partial relevance, and 1 study with unclear relevance. 9 studies total from 8 countries, including 3 high income, 3 middle income and 2 low-income countries. Geographical spread: 2 studies in Asia, 2 studies in Europe, 2 Africa, 2 studies in South America. The finding is highly relevant to the review question. The contributing studies are from diverse international settings, making the theme applicable to a broad range of contexts.	High confidence	9 studies with low methodological limitations. Data from 8 countries across all geographical regions. No or very minor concerns about coherence and adequacy.
2. Family Participation is a Necessity	(23),(24)	Low methodological limitations The contributing studies (23, 25) were assessed as having high methodological quality.	Minor concerns about coherence. The finding is coherent, with data from the primary studies consistently highlighting the crucial role of families in pediatric pain management.	Moderate concerns. The data supporting this finding, while rich, are drawn from a smaller number of studies within the synthesis. More data from a wider range of the included studies would increase the adequacy.	Minor concerns. The finding is highly relevant to the review question and has broad applicability.	Moderate confidence.	Two studies with high methodological quality. Minor concerns about coherence.
3. Evidence-Based vs. Experience-Based Pain Assessment	(25), (26)	Low methodological limitations The contributing studies (19, 22) was assessed as having high methodological quality.	Minor concerns. The finding is coherent, effectively capturing the tension between adherence to evidence-based guidelines and reliance on clinical experience.	Moderate concerns. The finding is predominantly supported by two rich study (22, 19). While the data are detailed, the lack of broader representation across the other included studies is a limitation.	Minor concerns. The finding is highly relevant to the review question.	Moderate	7 studies with Low methodological limitations, 2 studies with high methodological quality. Minor concerns about coherence.
4.Pharmacological vs. Non-Pharmacological Interventions	(23),(27)	Low methodological limitations The contributing studies (23, 25) were assessed as having high methodological quality.	Minor concerns. The finding is coherent, clearly outlining the challenges and preferences related to both pharmacological and non-pharmacological interventions.	Minor concerns. The data are rich and drawn from several studies. However, more details on the specific types of non-pharmacological interventions used across all included studies would improve adequacy.	Minor concerns. The finding is highly relevant to the review question.	High	7 studies with Low methodological limitations, 2 studies with high methodological quality. Minor concerns about coherence.
5. Culture-Related Pain Management	(21),(25)	Low methodological limitations. The contributing studies (21, 22) were assessed as having high methodological quality.	Minor concerns. The finding is coherent, with compelling data illustrating the influence of cultural beliefs on pain management.	Moderate concerns. The data are rich but are drawn from a limited number of cultural contexts within the synthesis (primarily Ethiopia and Indonesia). More data from the other included studies would be needed to support the breadth of this finding fully.	Minor concerns. The finding is highly relevant, but the assessment of relevance is somewhat constrained by the limited number of cultural contexts explored in depth.	Moderate	7 studies with Low methodological limitations, 2 studies with high methodological quality. Moderate concerns about the adequacy. Minor concerns about coherence.

## Results

### Studies included

The initial search produced 1,436 records (896 after duplicate removal). After the title and abstract reading, 861 records were excluded, and 35 underwent the eligibility process. After reading the full texts, 26 studies were excluded for different reasons (Figure 1 page 6), and a total of 9 studies were included (19–26) (Table 1).

### Characteristics of the studies

The studies included only nurses as participants. With sample sizes ranging from *n* = 6 to *n* = 37. Most participants were female (e.g., 30/37 in one study; 24/28 in another), with ages spanning 24–60 years. Experience levels varied widely: some studies required a minimum of 3–5 years in clinical practice, while others reported averages of 5 years (range: 6 months-24 years). Educational attainment included diplomas (*n* = 10–16), bachelor's (*n* = 5–15), and master's degrees (*n* = 2–4). Marital status was noted in one study (single: 5; married: 14). Specialty areas included pediatric wards (e.g., PICU, maternal, child), VIP units, and ICUs, with some participants holding leadership roles (e.g., 6 team leaders). While one included study featured a multidisciplinary ICU team (including physicians, total sample *n* = 30), our analysis exclusively examined nurses' perspectives in this study to maintain methodological focus, while others focused exclusively on nurses in pediatric or general hospital settings. The characteristics of the 9 included studies are summarized in Table 1.

### Quality appraisal of the included studies

Table 4 presents the quality appraisal of the nine included studies using the Critical Appraisal Skills Programme (CASP) checklist (28). Overall, the studies demonstrated strong methodological rigor in several key areas. All nine studies clearly stated their research aims, employed appropriate qualitative methodologies, utilized suitable research designs to address their aims, and adopted appropriate recruitment strategies. These consistent strengths indicate a foundational level of quality across the body of evidence.

**Table 4:** Quality Appraisal of the Included Studies Using the CASP^a^

Item	Mediani HS. (2017)	Aziznejadroshan. (2018)	Berihun B. (2024)	Susanto H. (2022)	D. Parra-Giordano. (2020)	A. Kusi Amponsah. (2020)	M. S. Silva. (2011)	A. Bhattacharyya. (2024)	Skog N. (2020)
Item 1. Was there a clear statement of the aims of the research?	Y	Y	Y	Y	Y	Y	Y	Y	Y
Item 2. Is a qualitative methodology appropriate?	Y	Y	Y	Y	Y	Y	Y	Y	Y
Item 3. Was the research design appropriate to address the aims of the research?	Y	Y	Y	Y	Y	Y	Y	Y	Y
Item 4. Was the recruitment strategy appropriate to the aims of the research?	Y	Y	Y	Y	Y	Y	Y	Y	Y
Item 5. Were the data collected in a way that addressed the research issue?	Y	Y	Y	Y	CT (sample size is too small)	CT (interview time is only 10-30 minute)	N (guiding question addressed pain perception. but did not explicitly barriers to management (the research topic).	Y	CT (interview time is only 16-26 minute)
Item 6. Has the relationship between researcher and participants been adequately considered?	Y	Y	Y	Y	Y	Y	Y	Y	Y
Item 7. Have ethical issues been taken into consideration?	Y	Y	Y	Y	Y	Y	Y	Y	Y
Item 8. Was the data analysis sufficiently rigorous?	Y	Y	Y	Y	Y	Y	Y	Y	Y
Item 9. Is there a clear statement of findings?	Y	Y	Y	Y	Y	Y	Y	Y	Y
Item 10. How valuable is the research?	Val	Val	Val	Val	Val	Val	Val	Val	Val

^a^ Data evaluated using the critical appraisal skills programme (CASP)^11^ CT=cannot tell; N=no; Val=valuable; Y=yes.

However, some variability was observed in how data were collected to address the research issue (CASP Item 5). While five studies explicitly demonstrated that their data collection methods directly addressed the research question (19–21, 25), two studies indicated that it was difficult to determine if the data collection fully addressed the research issue (22, 29). One study explicitly stated that the data collection did not fully address the research issue (27), and another study's data collection was noted to have a sample size that was too small (21). This nuanced appraisal suggests that while the studies generally met high standards for research design and aim clarity, there were occasional limitations in the depth or breadth of data collection in a few instances. These limitations should be considered when interpreting the findings, particularly for studies where data collection was noted as “cannot tell” or “no” for addressing the research issue. A detailed methodological quality assessment, including CASP scores, is provided in Table 4

### Meta-Synthesis outcomes

This systematic review revealed five interrelated themes that collectively shape nurses' experiences with pediatric pain management. The foundational theme, “Defeated by Pain,” captured nurses’ struggles with systemic barriers, including understaffing and resource constraints. Building on this, “Family as the Gatekeeper” emerged as a critical factor, demonstrating how parental involvement influences clinical decision-making. While evidence-based guidelines were theoretically available, our analysis uncovered significant variability in practical implementation across clinical settings. Notably, pharmacological interventions-maintained dominance despite increasing recognition of complementary non-pharmacological approaches. Most critically, cultural factors were found to fundamentally influence every dimension of pain assessment and therapeutic intervention. These interconnected themes illuminate the persistent barriers and potential pathways for improving pediatric pain care practices.

1. Being Defeated by Pain

Being defeated by pain represents a profound professional and existential crisis for nurses, characterized by persistent perceived failures in alleviating pediatric pain despite their clinical expertise. This phenomenon emerges at the intersection of multiple compounding factors: systemic constraints (including excessive workload and resource scarcity), professional limitations (such as restricted autonomy in decision-making and variable competency levels), and psychological distress. These challenges are further amplified by structural determinants, particularly workforce shortages leading to unsustainable nurse-patient ratios, hierarchical care models that limit nursing autonomy, and institutional resource deficits encompassing pharmacological shortages and inconsistent implementation of pain management protocols.

Inadequate nurse-to-patient ratios significantly compromise effective pain management delivery, adversely affecting both nursing professionals and pediatric patients. This staffing constraint systematically undermines nurses' capacity to fulfill their clinical responsibilities while maintaining quality care standards (20). As one nurse explained, “I can”t focus on caring for pediatric patients… I have many tasks to complete on this shift… I am not able to deliver effective pain care.” (20), This highlights how structural limitations directly translate into clinical and professional challenges. The imbalance between nursing resources, patient volume, and workload demands precipitates systemic failures in pain management protocols. This unsustainable situation compels nurse managers to implement clinically questionable staffing decisions and maintain exhausting schedules that ultimately degrade care quality (25). As one nurse stated, “We haven't enough time to use pain-assessment tools for large number of child” (25). While a manager admitted, “The severity of staff shortage was to the extent that I have to dedicate morning and afternoon shifts with no day-off to the monthly work schedule of all nurses, regardless of their experience.” (25).

The delivery of effective pain management is further compromised by systemic constraints on nursing autonomy, perpetuated through hierarchical medical dominance, limiting nurses' clinical decision-making authority despite their frontline expertise. This power imbalance becomes particularly evident when nurses must obtain physician authorization even for routine analgesic adjustments, as institutional policies systematically designate pain management as an exclusively physician-led domain. Participant noted, “As a nurse, you can do your part… It's not us who prescribe drugs, so mostly yours are therapeutic methods like diversion therapy unless you call doctors for them to give you the go-ahead… You can't get up and give a drug without the doctor's advice. So, everything you have to consult” (29). These professional constraints intersect with and are exacerbated by broader systemic failures, including deficient clinical guidelines and resource inadequacies. Another participant emphasized this compounding effect: “The tools for measuring pain are not available on the ward. Moreover, we do not have standard procedures for pain assessment and management… Many nurses do not know how to do a pain assessment theoretically… We are working without guidelines.” (20).

The persistent inadequacy in nurses' preparation for pediatric pain management stems from systemic educational gaps that fail to develop three critical competencies: evidence-based pain assessment, therapeutic intervention strategies, and effective patient advocacy skills. This knowledge deficit is poignantly captured in one nurse's admission: “As long as I have been working in this hospital, I have never attended a course or training about pain management because the hospital has not provided training for pain management in children. I have only basic knowledge based on my education and experience, and I am aware that my knowledge of pain assessment and pharmacological intervention is still limited.” (20) When combined with chronic staff shortages, restricted clinical autonomy, and resource insufficiencies, these educational deficiencies create a compounded crisis in pediatric pain management that spans institutional, professional, and clinical dimensions.

2. Family participation is a necessity

Family participation is a necessity in pediatric pain management, with families serving dual critical roles as gatekeepers of pain and key providers of continuing care. Parental involvement in pediatric care maintains developmental continuity by preserving familiar routines and relationships during hospitalization. This continuity, in turn, facilitates smoother transitions to home care, with research indicating a correlation to reduced preventable readmissions (30). Moreover, the synergistic relationship between nursing staff, parents, and patients has been empirically demonstrated to improve both objective clinical outcomes and subjective measures of humanized care across multiple healthcare settings. As one healthcare provider emphasized, “The emotional support provided by the family is, but I think it is key to pain management because deep down, you can give a child many drugs, but if the family is not supportive, that child will not be there. quiet no more." (26)

Families constitute the fundamental nexus of continuing care, establishing a vital bridge between formalized healthcare institutions and patients' lived experiences. A comprehensive meta-synthesis of qualitative evidence reveals that family caregivers fulfill multifaceted roles that integrate physical care delivery, psychosocial support, and healthcare system mediation (31). This continuity of therapeutic engagement transcends institutional parameters, as families implement and maintain consistent care approaches throughout transitions spanning hospital environments, and outpatient services, and domestic settings. The coordination of care demonstrates measurable improvements in patient health outcomes while simultaneously promoting psychological resilience during periods of clinical instability. As family caregivers navigate the complexities of the healthcare system, their involvement becomes crucial in ensuring that patients receive holistic and personalized care that addresses medical and emotional needs. The essential role of families as informational conduits is highlighted by one participant's observation, “Very often, companions report us that children are feeling pain at that moment (E10).” (27) Through these interconnected functions as both gatekeepers and continuity providers, families emerge as indispensable partners in effective pediatric pain management across the entire care continuum.

3. Evidence-Based vs. Experience-Based Pain Assessment

Evidence-based practice demonstrably enhances the delivery of safe, efficacious, and patient-centered care across clinical domains. Despite the substantial accumulation of research on pain assessment and intervention strategies spanning three decades, the implementation of evidence-based pain management continues to be mediated by the interplay between individual clinician factors (including knowledge deficits, attitudinal barriers, and practice preferences) and systemic organizational variables (such as resource allocation, policy frameworks, and institutional priorities) (32).

Nursing staff in the PICU and general wards adhered to institutional pain management protocols, particularly in conducting timely and evidence-based pain assessments. Compliance with these guidelines was driven by the belief that systematic assessment is critical for mitigating postoperative complications in pediatric patients. Assessment is number one…, we must do it for every patient here. Whether there is pain or anxiety. It may be visible in the patient's hemodynamics on the monitor. Also, nurses keep asking the patients whether they have pain or not, because that is the basis for taking further steps. During emergency conditions, we automatically give analgesics to reduce pain. (N1, PICU) (24). Nurses often defaulted to unit-specific pain scales rather than all guideline-recommended tools, though some reported using dual scales to cross-validate scores in uncertain cases. “First, if patients verbally reported pain, I would use the Wong-Baker FACES scale. However, if the score appeared inconsistent with their reported severity (e.g., low score but severe complaints), I would cross-validate using the COMFORT scale to ensure accuracy (N12, PICU).” (24)

Despite the availability of institutional guidelines, some nurses relied primarily on experiential knowledge rather than evidence-based protocols when assessing pediatric pain. These nurses prioritized subjective, experience-driven evaluations over standardized tools, relying on their clinical expertise (22), they often assessed pain levels based on prior experience rather than standardized tools. Many expressed confidence that prolonged exposure to pediatric cases enabled them to accurately recognize pain without relying solely on formal assessment protocols. As one pediatric nurse explained: “We do not really go by the guidelines. Maybe because we are used to pain issues in our unit. When we see a patient crying a little, we have a feeling that this patient has mild pain. If the patient cries a bit louder, we categorize it as moderate pain. If the patient screams and the parent is unable to calm the patient, we categorize it as severe pain. That's what I do based on my experience. I care for child patients very often (N7, Ped).” (24, 27)This preference for heuristic methods over protocolized assessments highlights a significant gap between evidence-based recommendations and bedside practice.

4. Pharmacological vs. non-pharmacological interventions for pain management:

Our study revealed that pediatric inpatient pain management relied heavily on pharmacological interventions, particularly for disease-related symptoms. While these drug-based protocols established a structured therapeutic framework, nurses highlighted significant implementation challenges in clinical practice. Of particular concern was the prevalent and often indiscriminate use of opioids, which frequently led to observable dependency patterns. Clinical staff emphasized the necessity of comprehensive patient assessment and careful consideration of subjective pain experiences when determining appropriate interventions. Although pharmacological prescriptions fall under physician authority, nurses maintain complete professional responsibility for administering medications, including dosage timing and delivery methods. As one participant noted: “They gave an analgesic therapy scheme as per the WHO, in general here, and depending on that, if it is well relieved .. it escalates in opiates already when it does not yield much and escalates until there is no pain” (26). This account illustrates the tension between standardized protocols and the nuanced clinical judgments required in pediatric pain management.

Non-pharmacological interventions represent an essential dimension of pediatric pain management, providing developmentally appropriate, evidence-based strategies that address pain through psychological, physical, and sensory mechanisms. these interventions constitute an essential component of effective pain management, it includes active listening, environmental modification, therapeutic accompaniment, and complementary therapies, all of which facilitate the establishment of a meaningful nurse-patient relationship crucial for optimal pain relief. Furthermore, nurses emphasize self-awareness as a foundational element of person-centered care, prioritizing therapeutic trust and interpersonal connection over technical procedures alone. As the nurse described: “When one manages to have an effective therapeutic relationship, and that is only by doing humanized care, they will have full confidence in you and also that you will be able to help them, and that reduces the burden of anxiety on the child. (E1)” (26). On the other hand, physical comfort measures, including therapeutic touch, massage, and positioning adjustments, are frequently employed non-pharmacological interventions. These approaches enhance patient comfort and psychological security while reducing stress and fear responses. Such strategies not only facilitate pain relief but also strengthen the therapeutic alliance between pediatric patients and nursing staff. “We caress, touch their fronts, change children's position in bed, or even cuddle them, swing, ask the companion, or even we bathe them to see whether they calm down. We may play, take some toys to call the children's attention (E8). (27) These practices illustrate how frontline providers operationalize patient-centered care through diverse non-pharmacological modalities.

5. Culture-related pain management

Cultural determinants of pain management emerged as a salient theoretical construct within the meta-synthesis, demonstrating the multidimensional influence of sociocultural contexts on nurses’ cognitive frameworks, assessment methodologies, and therapeutic interventions in pediatric pain management. Synthesized findings revealed that healthcare providers consistently encounter epistemological and praxis-oriented challenges stemming from culturally mediated variations in pain expression phenomenology (e.g., normative stoicism in collectivist communities vs. externalized distress behaviors in individualist societies), institutional decision-making hierarchies, and differential levels of ontological trust in biomedical paradigms.

The sociocultural positioning of families within healthcare systems serves as a critical mediator of analgesic efficacy through complex, intersecting pathways of influence. These pathways include factors such as cultural beliefs about pain, communication styles between healthcare providers and patients, and the availability of resources that support pain management. Understanding these dynamics is essential for developing tailored interventions that enhance analgesic effectiveness across diverse populations. A fundamental tension emerges between culturally diverse health belief systems and standardized biomedical protocols, creating substantial barriers to effective pain management across heterogeneous populations. This conflict is vividly illustrated when the nurse said “Coincidentally, it was months ago, and the child had a high fever. I was about to give IM anti-pain. Family will not give it, they said we would not inject it because it would be “evil eye/ቡዳ/” and then explain the problem to them if they agree. Just as I was injecting him, there was a delay in explaining them, so he fainted and started shaking, which is what we call a febrile seizure. From that family, they directly contacted with an injection and an evil eye, and while we were talking, they said that our son had killed him. There was a lot of controversy and they thought of something else…” (19) This case demonstrates how deeply entrenched cultural interpretations of illness and treatment can directly conflict with biomedical interventions, potentially compromising both clinical outcomes and therapeutic alliances.

In contrast to culturally mediated care barriers, spiritual beliefs fundamentally reshape the phenomenology of pain, imbuing nociceptive signals with transcendent meaning. Within pediatric critical care settings, spiritual and religious interventions have gained recognition as vital complementary modalities for managing pain and distress. Emerging evidence indicates that faith-based practices are systematically incorporated into holistic care frameworks, functioning simultaneously as adjunct therapeutic interventions and psychological support systems for pediatric patients. These approaches, encompassing both formal religious observances and personalized spiritual care, are routinely combined with biomedical treatments to address the biopsychosocial complexity of pain in children. As one PICU nurse described, “A patient's family once gave me prayed water (Water a cleric prayed over) and asked me to use it to bathe the patient's body, so we just did it, so that the family would be more satisfied. When giving the intervention (bathing the patient with prayed water), we recited al-Fatihah (part of the holy Quran)“. (27)

## Discussion

This meta-synthesis of nine qualitative studies examining nurses’ perspectives on pediatric pain management reveals a complex interplay of clinical, organizational, cultural, and interpersonal factors that collectively shape pain management practices. The five identified themes-Being Defeated by Pain, Family Participation is a Necessity, Evidence-Based vs. Experience-Based Pain Assessment, Pharmacological vs. non-pharmacological interventions for pain and Culture-Related Pain Management, offer a multidimensional framework for understanding the challenges and opportunities in optimizing pediatric pain care.

The foundational theme of “Being Defeated by Pain” exposes a critical dissonance between institutional protocols and frontline realities in pediatric pain management. Although healthcare systems formally endorse evidence-based pain management principles, our synthesis identifies significant implementation barriers rooted in systemic resource constraints and institutional prioritization. This aligns with Argyris and Schön's (1974) theory of organizational learning, where “espoused theories” often diverge from “theories-in-use” in professional practice (33). This gap corroborates findings from a multisite study highlighting “*low clinical priority accorded to pain management by medical teams”* as a persistent challenge in pediatric settings (34). Persistent staffing shortages and unsustainable nurse-patient ratios systematically compromise the delivery of optimal pain management, creating a disparity between institutional commitments to evidence-based practice and actual clinical realities. Organizational theorists term the chasm between formal policies and enacted practices in complex healthcare systems the “theory–practice gap.” (35). This moral distress has been associated with burnout and staff turnover, suggesting that resolving these systemic contradictions requires not just individual resilience training but structural reforms including nurse-inclusive policy design, protected time for pain assessments, and accountability metrics that prioritize pain management as a core quality indicator. This theme highlights how workload and organizational pressures undermine systematic pain assessment. Institutions should therefore shift from episodic pain checks to integrated, multidimensional assessment protocols embedded in the electronic health record (EHR), consistent with IASP(International Association for the Study of Pain) guidance and using age-appropriate, validated tools such as FLACC for non-verbal children (36).

Beyond the institutional and provider-centered barriers, our analysis identified the families as critical stakeholders whose influence fundamentally shapes pain management outcomes. The theme “Family participation is a necessity” repositions families from passive observers to active co-producers of care with determinative influence over pain trajectories. Our identification of families as “gatekeepers of pain” advances theoretical understanding by delineating their threefold mediating role: (1) therapeutic access control, (2) phenomenological interpretation, and (3) psychosocial containment.Families actively shape pain management through their continuous presence and intimate knowledge of the child's baseline behaviors. while their emotional regulation role during hospitalization reflects Bowlby's attachment theory, wherein secure attachment figures modulate distress in threatening contexts (37). Beyond immediate pain management, our synthesis positions families as pivotal agents of continuing care, creating a vital bridge between institutional healthcare delivery and patients’ lived experiences.. Their sustained therapeutic engagement extends Meleis et al.'s (2000) transitions theory, demonstrating how families function as “continuity agents” during health-illness transitions (38) These findings advocate for clinical protocols that formally integrate family-led pain assessments into electronic documentation systems, ensuring their experiential knowledge is preserved across care transitions.

Empirical evidence corroborates this role, linking active family engagement to both reduced hospital readmissions and improved treatment adherence (39). To operationalize this shift, healthcare institutions must redesign interprofessional training to prioritize shared decision-making and adjust nurse staffing models to accommodate family collaboration time, addressing both the “theory-practice gap” and systemic workload barriers identified in our synthesis. Building on these findings, families act as “gatekeepers” and key partners in pain detection, emphasizing the need to formalize family-led assessment. Clinical protocols should incorporate parental observations into individualized pain management plans, and nurses should be trained in proxy-report instruments that allow parents to document behavioral indicators of pain (40). A structured parental pain observation tool, used alongside existing clinical scales, can provide a more comprehensive interpretation of the child's distress, especially when clinical signs are unclear.

The meta-synthesis reveals that while evidence-based protocols exist, their integration into daily practice remains inconsistent and challenging. This aligns with the Star Model of Knowledge Transformation, which posits that moving from discovery to practice implementation requires navigating multiple barriers at individual, team, and system levels (41). The identified challenges in implementing evidence-based pain assessment tools echo findings from study results, which showed significant gaps between recommended and actual pain assessment practices in pediatric settings (42). When evidence-based pain management practices conflict with established workflows or institutional priorities, they struggle to achieve normalization despite their demonstrated efficacy. Interestingly, our findings suggest that implementation challenges vary across different dimensions of pain management. Assessment practices appear particularly vulnerable to implementation barriers, perhaps reflecting what was identified as the “invisibility” of assessment work—clinical labor that remains largely unrecognized and undervalued within healthcare systems (43). This invisibility may explain why, despite robust evidence supporting systematic pain assessment, it remains inconsistently practiced in many pediatric settings.

Turning to pharmacological pain management approaches, our synthesis reveals a nuanced tension between standardized protocols and individualized care. While nurses value structured analgesic frameworks (such as the WHO pain ladder), they simultaneously emphasize the importance of personalized approaches based on individual patient assessment. This tension reflects broader debates in healthcare regarding the appropriate balance between standardization and customization (44). The findings regarding opioid administration practices are particularly significant given contemporary concerns about opioid use. Our synthesis also highlights the complex role of boundaries in pharmacological pain management, where prescription falls under medical authority while administration timing and method remain nursing responsibilities. The effectiveness of pharmacological pain management thus depends not only on evidence-based protocols but also on successful interprofessional collaboration and communication. Complementing pharmacological approaches, the emergence of non-pharmacological interventions as a critical component of pediatric pain management reflects a growing recognition of pain's multidimensional nature. Our findings demonstrate that nurses actively employ diverse psychological, physical, and sensory modalities to address pain. This aligning with the biopsychosocial model of pain that has gained prominence in recent decades (45). This approach represents a significant evolution from earlier biomedical models that focused primarily on nociceptive and pharmacological management. However, our findings extend beyond efficacy to illuminate how these approaches are implemented in clinical practice, revealing both creative adaptations and implementation challenges. Particularly noteworthy is the emphasis on minimizing iatrogenic risks through non-pharmacological approaches. The paradigm shifts toward integrative care identified in our synthesis thus represents not merely an expansion of available interventions but a fundamental reconceptualization of what constitutes optimal pain management, defining success in terms of both physiological relief and psychosocial well-being.

Among the most theoretically rich aspects of our meta-synthesis are the findings concerning the cultural dimensions of pediatric pain management. Findings regarding culturally mediated variations in pain expression echo research that documented significant cultural differences in pain behaviors and reporting patterns among children (46). When healthcare providers’ biomedical explanatory models conflict with families’ culturally constructed health belief systems, the result can be what our synthesis terms “suboptimal symptom reporting behaviors and diminished therapeutic adherence.” Particularly significant is the identification of spiritual beliefs as reconfiguring “the very phenomenology of pain, transforming nociceptive into an experience laden with transcendent significance.” This finding extends beyond cultural variation in pain expression to encompass fundamental ontological differences in how pain is conceptualized and experienced. The incorporation of faith-based practices alongside conventional medical interventions represents what was described as the integration of spirituality into holistic healthcare, an approach that acknowledges the multidimensional nature of human suffering and healing (47).

## Conclusion

This meta-synthesis illuminates the complex interplay of factors shaping pediatric pain management from nurses’ perspectives. The identified themes, systemic contradictions, implementation challenges, pharmacological approaches, non-pharmacological interventions, and cultural dimensions collectively demonstrate that optimal pain management requires attention not only to clinical evidence but also to organizational contexts, interprofessional dynamics, and cultural considerations. These insights can guide healthcare professionals in implementing evidence-based strategies, enhance curriculum development for future practitioners, and inform policymakers in creating supportive frameworks that promote effective health interventions. Clinically, findings advocate for balanced approaches integrating standardized protocols with individualized assessment, recognizing that optimal pain management requires both structured frameworks and personalized adaptations. For nursing education, results emphasize developing competencies beyond technical skills to include cultural competence, and interprofessional collaboration essential for addressing pediatric pain management's multifaceted challenges. In addition, healthcare providers should develop collaborative strategies with families that leverage families’ unique knowledge of children's behaviors and engage with families as partners. Despite valuable insights, limitations include potential obscurity of context-specific factors due to diverse healthcare settings and the exclusive focus on nurses’ perspectives. Future research directions should explore additional stakeholders’ experiences (particularly children's), conduct longitudinal studies examining practice evolution, and test interventions addressing identified systemic contradictions and implementation barriers.

### Strengths and limitations

This meta-synthesis offers an in-depth integration of nurses' perspectives on pediatric pain management, but several limitations must be considered. First, it draws on only nine primary studies, which may limit the robustness and generalizability of the synthesized themes. Although thematic saturation was observed, the small evidence base means that some nuanced views or rare clinical situations may not be represented. Second, the studies are geographically clustered (e.g., China, Ethiopia, Europe), which constrains the transferability of the findings. Because pain and its management are culturally shaped, the overrepresentation of certain regions may overemphasize some cultural barriers while under-representing others. Third, the included studies reflect diverse healthcare systems, from resource-limited settings to highly specialized units. This contextual variability affects nurses' experiences of autonomy and resources, making it difficult to recommend a single, universally applicable strategy for improving pain management. Finally, the restriction to English-language publications may have introduced publication and language bias. Excluding non-English studies likely omitted important perspectives from non–English-speaking regions and may have reduced the cultural breadth of insights into pediatric pain care. Future research should incorporate multi-lingual searches to enhance global representativeness and inclusivity (48).
